# Generalized roughness of three dimensional ($\in ,\in \vee q$)-fuzzy ideals in terms of set-valued homomorphism

**DOI:** 10.1038/s41598-024-62207-8

**Published:** 2024-05-29

**Authors:** Shahida Bashir, Rabia Mazhar, Nasreen Kausar, Saziye Yaman, Syed Suleman Ali, Muneeb Ul Hassan Afzal

**Affiliations:** 1 Department of Mathematics, University of Gujrat, Gujrat, 50700 Pakistan; 2 Department of Mathematics, Faculty of Arts and Science, Yildiz Technical University, 34220 Esenler, Istanbul Turkey; 3 Department of Liberal Arts, American University of the Middle East, 54200 Egaila, Kuwait; 4 Department of Mechanical Engineering, University of Gujrat, Gujrat, 50700 Pakistan

**Keywords:** Ternary semiring, $(\in ,\in \vee q)$-fuzzy ideal, Lower and upper approximations, Set-valued homomorphism, Strong set-valued homomorphism, Engineering, Mathematics and computing

## Abstract

The objective of this study is to generalize the roughness of a fuzzy set-in three-dimensional structure by introducing ternary multiplication. Many results and theorems of rough fuzzy ideals have been extended from semigroup and semiring, to ternary semiring by introducing the definition of a rough fuzzy subset of ternary semiring. By using the concept of set-valued homomorphism and strong set-valued homomorphism, it is proved generalized lower and upper approximations of $(\in , \in \vee q)$-fuzzy ideals (semiprime and prime ideals) of ternary semirings are $(\in ,\in \vee q)$-fuzzy ideals (semiprime and prime ideals) respectively.

## Introduction

Some algebraic structures are not closed using the binary operation of multiplication. For example, the set of positive integers ${Z}^{+}$ is closed with respect to the binary operation of multiplication. The closure law does not hold in the set of negative integers ${Z}^{-}$ with respect to the binary product, but holds with respect to the ternary product. To address these problems, a ternary operation was introduced. In 1932, the ternary algebraic structure was first initiated by Lehmer^1^. In 2003, the theory of ternary semiring was proposed by Kar and Dutta, who studied its characteristics as simplifications of ternary rings^2,3^. Kavikumar et al. worked on ternary semirings and their ideals, bi-ideals, and quasi-ideals subjected to fuzzy sets and it is continuous, along with many other properties^4^. For more work on ternary structures see Refs.^5–7^.

Algebraic patterns are very important in mathematics and are used in engineering, physics, computers, coding, topological space, automata theory, formal language, modelling, graph theory^8–10^. Vandiver introduced an algebraic system that consists of a non-empty set $R$ with two binary operations addition $(+)$ and multiplication $(\cdot )$, where $R$ is a semigroup under both operations. Framework $(R,+,\cdot )$ hold distributive laws under $(\cdot )$ over $(+)$^11^. Examples of semirings include natural numbers ℕ, positive real numbers and positive rational numbers under usual multiplication and addition. None of them are rings.

In many scientific fields, information may be uncertain and complex. In classical mathematics, all mathematical techniques and formulas are exact, such as a crisp set in which the characteristic function gives only two values, '$1$' and '$0$'. These values give only '$Yes$' and '$No$' responses respectively. With this formulation, we cannot deal with problems with uncertainty. After many efforts, tools have been introduced to address such problems. The fuzzy set theory is one of them, introduced by Zadeh to handle such conditions^12^. The characteristic function is mapped to the interval [0, 1], where the boundary value 0 indicates no affiliation to the fuzzy set and 1 indicates affiliation to the fuzzy set. whereas (0, 1) implies partial belonging to the fuzzy set. Later, many researchers presented a large number of characterizations and properties of fuzzy set^13–16^. Ming introduced the idea of a fuzzy point, its membership and its quasi-incidence $(q)$ on a fuzzy set^17^. $(\alpha ,\beta )$ -fuzzy subgroups were defined by Das and Bhakat^18^. These structures are restricted to substructures of $(\alpha ,\beta )$ -fuzzy semirings where $\alpha ,\beta$ are equal to $\in ,q,\in \vee q$ or $\in \wedge q$ while $\alpha \ne \in \wedge q$^19^.

**Figure 1:**
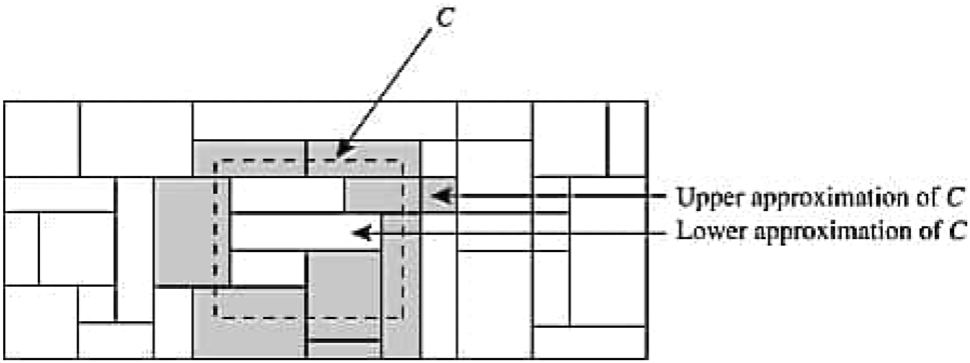
Upper and lower approximations.

Another theory that deals with uncertain problems is the rough set theory. This approach was first defined by Pawlak in 1982^20^. To some extent, it is vital to encounter ambiguous, unclear, and inexact data. Many researchers have been magnetized to learn the rough set approach and its use. We cannot oversee the approximations when discussing rough sets. The corresponding relationships are critical for fabricating approximations in Pawlak rough sets. A graphical representation of the rough set is shown in Fig. 1, which helps to understand the definition of a rough set. This set is characterized by lower and upper approximations. For universe $W$ and $C\subset W,$ the approximations of $C$ are given in Fig. 1. Here each rectangle defines an equivalence class, and the equivalence classes are the small granularities in the complete data. In the same equivalence classes, entries are indiscernible. The equivalence classes that are completely enclosed in $C,$ are lower approximations. The upper approximations (grey rectangles) consist of all equivalence classes that partially belong to $C$ and the lower approximations.

The rough set approach is a definite solution in situations where incomplete details are found^21^. Iwinski, and Pomykala determined the algebraic properties of the rough sets^22,23^. In 2020, Bashir et al. studied the three-dimensional congruence relation of the rough fuzzy ideals of ternary semigroups^24^. Recent, Wang introduced the fundamental properties of fuzzy rough sets based on triangular norms^25^ and Ibrahim defined new extensions of fuzzy sets using rough topology^26^. Rameez et al. studied the roughness of $(\in ,\in \vee q)$-fuzzy ideals of hemirings^27^.

In 2022, Shabir et al. approximated fuzzy ideals of semirings using the bipolar fuzzy technique^28^. Recently, Bashir et al. studied the roughness in $(\in ,\in \vee q)$-fuzzy ideals of ternary semigroups using set-valued homomorphism^29^. In this study, we extend the structure of Rameez et al.^27^ and Bashir et al.^28^ for the ternary semirings.

The remainder of this paper is organized as follows. In Sect. “Preliminaries”, a few basic concepts are connected with ternary semirings, fuzzy sets, and rough sets. In Sect. "Approximations of fuzzy ideals in ternary semirings", the lower and upper approximations of fuzzy ternary subsemirings, fuzzy ideals, fuzzy semiprime ideals and fuzzy prime ideals of ternary semirings are studied using a set-valued homomorphism. Approximations of $(\in ,\in \vee q)$-fuzzy ternary subsemirings and $(\in ,\in \vee q)$-fuzzy ideals are worked in Sect. “[Sec Sec4]”. Finally, conclusions are presented. The acronyms used in this study, are listed in Table 1.

**Table 1:** List of acronyms.

Acronyms	Representation
SVH	Set-valued homomorphism
SSVH	Strong set-valued homomorphism

## Preliminaries

Ternary semiring is a non-empty set with two operations ' + ' (usual addition) and ' $$\cdot$$ ' (ternary product) such as ($$R,+)$$ is a commutative semigroup, ($$R,\cdot )$$ is a semigroup and distributive laws hold in $$R$$ i.e. $$((p+l)mn)=(pmn)+(lmn),$$ $$(p(l+m)n)=(pln)$$ $$+(pmn)$$ and $$(pl(m+n)=(plm)+(pln)$$ for all $$p,l,m,n\in R$$. By subset, we always refer to a non-empty subset. Throughout this paper, $$R$$ is the ternary semiring and $$\eta$$ is the fuzzy subset of $$R.$$ A subset $$S$$ of $$R$$ is called the subsemiring of $$R$$ if ($$S,+)$$ itself is closed, and $$SSS\subseteq S.$$ Subset $$M$$ of $$R$$ is called the right (resp. left, lateral) ideal of $$R$$ if ($$M,+)$$ is closed, and $$MRR\subseteq M$$ $$($$ resp. $$RRM\subseteq M,RMR\subseteq M)$$. If $$M$$ is the left, right and lateral ideal of $$R$$, then $$M$$ is called an ideal of $$R.$$ A proper ideal $$M$$ of $$R$$ is said to be a prime ideal of $$R$$ if $$IJK\subset M$$ implies $$I\subset M$$ or $$J\subset M$$ or $$K\subset M$$, for all ideals $$I,J,K$$ of $$R$$ and it is semiprime if $${I}^{3}\subset M$$ implies $$I\subset M$$ for all ideal $$I$$ of $$R.$$

### Definition 2.1

A fuzzy subset $\upeta$ of R is called a fuzzy point if $$\eta \left( m \right) = \left\{ {\begin{array}{*{20}c} {r {\text{if}} m = l} \\ {0 {\text{if}} m \ne l} \\ \end{array} } \right.$$ where ${l}$ is the support of $\upeta$ and ${r}\in [0, 1]$ is its value. The fuzzy point is expressed as ${{l}}_{{r}}$^30^.

### Definition 2.2

Suppose $\upeta$ is a fuzzy subset of ${R}$ and ${{l}}_{{r}}$ is a fuzzy point. Then, we can say ${{l}}_{{r}}$ belongs to (quasi-coincident with) $\upeta$ written as ${{l}}_{{r}}\in\upeta$ $({{l}}_{{r}}{q\eta })$ respectively as follows^30^:

1. *If *$\eta (l)\ge r,$* then it means *${l}_{r}$* belongs to *$\eta$* and is written as* ${l}_{r}\in \eta .$
2. *If *$\eta (l)+r>1$* then *${l}_{r}$* is said to be quasi-coincident with *$\eta$* and is denoted as* ${l}_{r}q\eta .$
3. *If *$\eta (l)\ge r$* or *$\eta (l)+r>1$* then *${l}_{r}$* belongs to *$\eta$* or *${l}_{r}$* is quasi-coincident with *$\eta$* and is denoted as *${l}_{r}\in \vee q\eta$*.*
4. *If *$\eta (l)\ge r$* and *$\eta (l)+r>1$* then *${l}_{r}$* belongs to *$\eta$* and *${l}_{r}$* is is quasi-coincident with *$\eta$* and is denoted as *${l}_{r}\in \wedge q\eta .$* When any of *${l}_{r}\in \eta ,{l}_{r}q\eta ,$* or *${l}_{r}\in \vee q\eta$* does not hold, then we write *${l}_{r}\overline{\in }\eta ,{l}_{r}\overline{q}\eta ,$* or *${l}_{r}\overline{\in \vee q}\eta$* respectively.*

### Definition 2.3

A fuzzy ternary subsemiring $\upeta$ of ${R}$ is defined as for all ${o},{p},{q}\in {R}$ as follows^30^:

1. $$\eta (o+p)\ge \mathit{min}\{\eta (o),\eta (p)\};$$
2. $$\eta (opq)\ge \mathit{min}\{\eta (o),\eta (p),\eta (q)\}.$$

### Definition 2.4

The fuzzy left (resp. right, lateral) ideal of ${R}$ is defined as for all ${o},{p},{q}\in {R}$ it satisfies^30^.

1. $$\eta (o+p)\ge \mathit{min}\{\eta (o),\eta (p)\};$$
2. $$\eta (opq)\ge \eta (q)$$ $$($$ resp. $$\eta (opq)\ge \eta (o)$$ ,$$\eta (opq)\ge \eta (p));$$

### Definition 2.5

A fuzzy ideal $\upeta$ of ${R}$ is called a semiprime if $\upeta ({rrr})\ge\upeta ({r})$ and prime if $\upeta ({opq})=\upeta ({o})$ or $\upeta ({opq})=\upeta ({p})$ or $\upeta ({opq})=\upeta ({q})$ for all ${o},{p},{q},{r}\in {R}$ (2) $\upeta ({opq})\ge\upeta ({q})$ $($resp. $\upeta ({opq})\ge\upeta ({o})$, $\upeta ({opq})\ge\upeta ({p}));$

### Definition 2.6

An $(\in ,\in \vee {q})$ -fuzzy ternary subsemiring $\upeta$ of ${R}$ is defined as $,$ for all ${l},{m},{n}\in {R}$ and ${r},{t},\updelta \in (0, 1]$^30^.

1. $${l}_{r},{m}_{t}\in \eta \to (l+m{)}_{\mathit{min}(r,t)}\in \vee q\eta ;$$
2. $${l}_{r},{m}_{t},{n}_{\delta }\in \eta \to (lmn{)}_{\mathit{min}(r,t,\delta )}\in \vee q\eta .$$

### Definition 2.7

An $(\in ,\in \vee {q})$—fuzzy left (resp. right and lateral), the ideal $\upeta$ of ${R}$ is defined as for all ${l},{m},{n}\in {R}$ and ${r},{t}\in (0, 1]$^30^.

1. $${l}_{r},{m}_{t}\in \eta \to (l+m{)}_{\mathit{min}(r,t)}\in \vee q\eta ;$$
2. ${l}_{r}\in \eta$ and $m,n\in R\to (mnl{)}_{r}\in \vee q\eta ,$ (resp. $(mln{)}_{r}\in \vee q\eta$ and $(lmn{)}_{r}\in \vee q\eta ).$

### Definition 2.8

If $\upeta$ is an $(\in ,\in \vee {q})$—fuzzy ideal of ${R}$ then it is called semiprime if for all ${l}\in {R}$ and ${r}\in (0, 1]$ such that $({{l}}^{3}{)}_{{r}}\in\upeta$ implies ${{l}}_{{r}}\in \vee {q\eta }.$

### Definition 2.9

If $\upeta$ is an $(\in ,\in \vee {q})$-fuzzy ideal of ${R}$ then it is called prime if for all ${l},{m},{n}\in {R}$ and ${r}\in ({0,1}]$ such that $({lmn}{)}_{{r}}\in\upeta$ implies ${{l}}_{{r}}\in \vee {q\eta },{{m}}_{{r}}\in \vee {q\eta },{{n}}_{{r}}\in \vee {q\eta }.$

### Theorem 2.10

If ${{l}}_{{r}},{{m}}_{{t}}\in\upeta \to ({l}+{m}{)}_{{min}({t},{r})}\in \vee {q\eta },$ then $\upeta ({l}+{m})\ge {min}\{\upeta ({l}),\upeta ({m}),0.5\}$ for all ${l},{m}\in {R}.$

### Proof

Let $l,m\in R,$ then we have two cases.

(i) $$\eta (l)\wedge \eta (m)\ge 0.5;$$
(ii) $$\eta (l)\wedge \eta (m)\le 0.5.$$

Contrarily suppose that there exist $l,m\in R$ such that $\eta (l+m)<\mathit{min}\{\eta (l),\eta (m),0.5\}\to (1)$.

Case (i) $\eta (l)\wedge \eta (m)\ge 0.5,$ then (1) implies $\eta (l+m)<0.5$ which implies that $(l+m{)}_{0.5}\overline{\in }\eta$.

Also, $\eta (l)\wedge \eta (m)\ge 0.5$ implies that $\eta (l)\ge 0.5$ and $\eta (m)\ge 0.5$ from this we get ${l}_{0.5}\in \eta$ and ${m}_{0.5}\in \eta .$

Similarly $(l+m{)}_{0.5}\overline{q}\eta$ because $\eta (l+m)+0.5\le 1$ so,$(l+m{)}_{0.5}\overline{\in \vee q}\eta .$

In case (ii) $\eta (l)\wedge \eta (m)\le 0.5,$ then (1) implies that $\eta (l)\wedge \eta (m)<\mathit{min}\{\eta (l),\eta (m)\}$ so thus $r\in ({0,0.5})$ exists such that $\eta (l+m)<r=\mathit{min}\{\eta (l),\eta (m)\}$ which implies $(l+m{)}_{r}\overline{\in }\eta$ and ${l}_{r}\in \eta ,{m}_{r}\in \eta$. Similarly, $(l+m{)}_{r }\overline{q}\eta$ because $\eta (l+m)+r<r+r<1$

So, $(l+m{)}_{0.5}\overline{\in \vee q}\eta .$ Therefore, for all $l,m\in R,$ $\eta (l+m)\ge \{\eta (l),\eta (m),0.5\}.$

### Theorem 2.11

If ${{l}}_{{r}},{{m}}_{{t}},{{n}}_{\updelta }\to ({lmn}{)}_{{min}({r},{t},\updelta )}\in \vee {q\eta }$ then $\upeta ({lmn})\ge \wedge \{\upeta ({l}),\upeta ({m}),\upeta ({n}),0.5\}$ for all ${l},{m},{n}\in {R}.$

### Proof

Let $l,m,n\in R,$ then we have two cases.

(i) $$\eta (l)\wedge \eta (m)\wedge \eta (n)\ge 0.5;$$
(ii) $$\eta (l)\wedge \eta (m)\wedge \eta (n)<0.5.$$

Contrarily suppose that for $l,m,n\in R,$ $\eta (lmn)<\wedge \{\eta (l),\eta (m),\eta (n),0.5\}\to (1)$

Case (i) $$\eta (l)\wedge \eta (m)\wedge \eta (n)\ge 0.5$$ then.

(1) implies $\eta (lmn)<0.5,$ then $(lmn{)}_{0.5}\overline{\in }\eta$ also, $\eta (l)\wedge \eta (m)\wedge \eta (n)\ge 0.5$ implies that $\eta (l)\ge 0.5,\eta (m)\ge 0.5,\eta (n)\ge 0.5$ therefore ${l}_{0.5}\in \eta ,{m}_{0.5}\in \eta ,{n}_{0.5}\in \eta$ but $(lmn{)}_{0.5}\overline{\in }\eta$ also $(lmn{)}_{0.5}\overline{q}\eta$ because $\eta (lmn)+0.5<1$ so, $(lmn{)}_{0.5}\overline{\in \vee q}\eta$ which is a contradiction.

Case (ii): $\eta (l)\wedge \eta (m)\wedge \eta (n)<0.5$ (1) $\Rightarrow \eta (lmn)<\{\eta (l)\wedge \eta (m)\wedge \eta (n)\}$ so there exist $r\in ({0,0.5})$

such that $\eta (lmn)<r=\{\eta (l)\wedge \eta (m)\wedge \eta (n)\}$ this implies $(lmn{)}_{r}\overline{\in }\eta$ and $\{\eta (l)\wedge \eta (m)\wedge \eta (n)\}=r$ gives $\eta (l)\ge r,\eta (m)\ge r,\eta (n)\ge r$ implies ${l}_{r}\in \eta ,{m}_{r}\in \eta ,{n}_{r}\in \eta$ but $(lmn{)}_{r}\overline{\in }\eta$ also $(lmn{)}_{0.5}\overline{q}\eta$ so our supposition is wrong.

Hence, $$\eta (lmn)\ge \wedge \{\eta (l),\eta (m),\eta (n)\}.$$

### Theorem 2.12

If $\upeta$ is $(\in ,\in \vee {q})$—fuzzy left (resp. right and lateral) ideal of ${R}$, then $\upeta ({mnl})\ge \wedge \{\upeta ({l}),0.5\}$ $($resp.$\upeta ({mnl})\ge \wedge \{\upeta ({m}),0.5\},\upeta ({mnl})\ge \wedge \{\upeta ({n}),0.5\})$.

### Proof

Follows from Theorem 2.10 and Theorem 2.11.

### Theorem 2.13

If $\upeta$ is an $(\in ,\in \vee {q})$-fuzzy semiprime ideal of ${R},$ then for all ${l}\in {R},$ $\upeta ({l})\ge \wedge \{\upeta ({{l}}^{3}),0.5\}$.

### Proof

The proof follows from Theorem 2.10 and Theorem 2.11.

### Theorem 2.14

If $\upeta$ is an $(\in ,\in \vee {q})$-fuzzy prime ideal of ${R}$ then for all ${l},{m},{n}\in {R},\vee \{\upeta ({l}),\upeta ({m}),\upeta ({n})\}\ge \wedge \{\upeta ({lmn}),0.5\}.$

### Proof

Follows from Theorem 2.10 and Theorem 2.11.

### Definition 2.15

Suppose $\upeta$ is a fuzzy subset of ternary semiring ${R}$. Then, the sets ${{L}}_{-}(\upeta )({s})=\wedge \{\upeta ({t})|\hspace{0.33em}{t}\in [{s}{]}_{{L}}\}$ and ${{L}}^{-}(\upeta )({s})=\vee \{\upeta ({t})|\hspace{0.33em}{t}\in [{s}{]}_{{L}}\}$ are the lower and upper approximations of $\upeta$ respectively.

### Definition 2.16

For every ${x}\in {R},$ we define fuzzy subsets $\underset{\_}{{S}(\upeta )}({l})=\underset{{n}\in {S}({l})}{\wedge }\upeta ({n})$ and $\overline{{S}(\upeta )}({l})=\underset{{n}\in {S}({l})}{\vee }\upeta ({n}).$ $\underset{\_}{{S}(\upeta )}$ is the lower approximation and $\overline{{S}(\upeta )}$ is the upper approximation of $\upeta$ with respect to mapping ${S}$. Combination $(\underset{\_}{{S}\left(\upeta \right)}, \overline{{S}(\upeta )})$ is called rough fuzzy subset of ${R}$ if $\underset{\_}{{S}(\upeta )}=\overline{{S}(\upeta )}.$

### Definition 2.17

Let ${R}$ and ${{R}}^{{^{\prime}}}$ be ternary semirings. A set-appreciated map ${S}\hspace{0.33em}\hspace{0.33em}:\hspace{0.33em}\hspace{0.33em}{R}\to {{P}}^{*}({{R}}^{{^{\prime}}})$ is called a set-valued homomorphism, if for all ${n},{m},{l}\in {R}$

1. $$S(n)+S(m)\subseteq S(n+m);$$
2. $$S(n)S(m)S(l)\subseteq S(nml)$$

### Definition 2.18

Let ${R}$ and ${{R}}^{{^{\prime}}}$ be ternary semirings. A set appreciated map ${S}\hspace{0.33em}\hspace{0.33em}:\hspace{0.33em}\hspace{0.33em}{R}\to {{P}}^{*}({{R}}^{{^{\prime}}})$ is called a strong set-valued homomorphism, if for all ${n},{m},{l}\in {R}$.

1. $$S(n)+S(m)=S(n+m);$$
2. $$S(n)S(m)S(l)=S(nml).$$

Here, ${P}^{*}({R}^{\prime})$ means the set of all non-empty crisp subset of ${R}^{\prime}.$

In this study, for all $m$ $\in R$, image $S(m)$ is always a non-empty subset of ${R}^{\prime}$. In addition, here $S$ is a natural mapping $S\hspace{0.33em}\hspace{0.33em}:\hspace{0.33em}\hspace{0.33em}R\to P(R)$; unless stated otherwise.

## Approximations of fuzzy ideals in ternary semirings

In this section, the approximations of fuzzy ternary subsemirings are studied. By theorems, it is proved that the lower and upper approximations of fuzzy ideals (semiprime and prime) are fuzzy (semiprime and prime) ideals, respectively. Here, we use the concepts of set-valued homomorphism (SVH) and strong set-valued homomorphism (SSVH).

### Theorem 3.1

If ${S}$ is an SSVH and $\upeta$ is a fuzzy ternary subsemiring of ${R}$, then $\underset{\_}{{S}}(\upeta )$ is a fuzzy ternary subsemiring of ${R}.$

### Proof

Since $\eta$ is a fuzzy ternary subsemring of $R$, then for all $l,m,n\in R$

$$\eta (l+m)\ge \eta (l)\wedge (m)$$ and $$\eta (lmn)\ge \eta (l)\wedge \eta (m)\wedge \eta (n)$$

Consider,

$$\begin{aligned} \underset{\raise0.3em\hbox{$\smash{\scriptscriptstyle-}$}}{S} \left( \eta \right)\left( {l + m} \right) & = \wedge _{{r \in S\left( {l + m} \right)}} \eta \left( r \right){\text{ since }}S{\text{ is a }}SSVH \\ &= \mathop \wedge \limits_{{r \in \left[ {S\left( l \right) + S\left( m \right)} \right]}} \eta \left( r \right){\text{ take }}r = q + h{\text{such that }}q \in S\left( l \right){\text{and }}h \in S\left( m \right) \\ &= \mathop \wedge \limits_{{\left( {q + h} \right) \in \left[ {S\left( l \right) + S\left( m \right)} \right]}} \eta \left( {q + h} \right) \\ & \ge \mathop \wedge \limits_{{\mathop {h \in S\left( m \right)}\limits^{{q \in S\left( l \right)}} }} \left\{ {\eta \left( q \right) \wedge \eta \left( h \right)} \right\} \\ &= \left\{ {\mathop \wedge \limits_{{q \in S\left( l \right)}} \eta \left( q \right)} \right\} \wedge \left\{ {\mathop \wedge \limits_{{h \in S\left( m \right)}} \eta \left( h \right)} \right\} \\ &= \underset{\raise0.3em\hbox{$\smash{\scriptscriptstyle-}$}}{S} (\eta )(l) \wedge \underset{\raise0.3em\hbox{$\smash{\scriptscriptstyle-}$}}{S} (\eta )(m). \\ \end{aligned}$$

This implies that, $\underline{S}\left(\eta \right)\left(l+m\right)\ge \underline{S}\left(\eta \right)\left(l\right)\wedge \underline{S}\left(\eta \right)\left(m\right)\to (1)$

Again consider,

$$\begin{aligned} \underset{\raise0.3em\hbox{$\smash{\scriptscriptstyle-}$}}{S} \left( \eta \right)\left( {lmn} \right) & = \mathop \wedge \limits_{{r \in S\left( {lmn} \right)}} \eta \left( r \right){\text{ }}\sin ce{\text{ }}S{\text{ }}is{\text{ }}SSVH \\ &= \mathop \wedge \limits_{{r \in S\left( l \right)S\left( m \right)S\left( n \right)}} \eta \left( r \right){\text{ }}take,\,\,r = pqh{\text{ }}such{\text{ }}that{\text{ }}p \in S\left( l \right),q \in S\left( m \right),andh \in S\left( n \right), \\ & = \mathop \wedge \limits_{{pqh \in S\left( l \right)S\left( m \right)S\left( n \right)}} \eta \left( {pqh} \right) \\ &\ge \mathop \wedge \limits_{{p \in S\left( l \right),q \in S\left( m \right),h \in S\left( n \right)}} \left\{ {\eta \left( p \right),\eta \left( q \right),\eta \left( h \right)} \right\} \\ &= \left\{ {\mathop \wedge \limits_{{p \in S\left( l \right)}} \eta \left( p \right)} \right\} \wedge \left\{ {\mathop \wedge \limits_{{q \in S\left( l \right)}} \eta \left( q \right)} \right\} \wedge \left\{ {\mathop \wedge \limits_{{h \in S\left( l \right)}} \eta \left( h \right)} \right\} \\ &= \underset{\raise0.3em\hbox{$\smash{\scriptscriptstyle-}$}}{S} (\eta )(l) \wedge \underset{\raise0.3em\hbox{$\smash{\scriptscriptstyle-}$}}{S} (\eta )(m) \wedge \underset{\raise0.3em\hbox{$\smash{\scriptscriptstyle-}$}}{S} (\eta )(n). \\ \end{aligned}$$

This implies, $$\underline{S}\left(\eta \right)\left(lmn\right)\ge \underline{S}\left(\eta \right)\left(l\right)\wedge \underline{S}\left(\eta \right)\left(m\right)\wedge \underline{S}\left(\eta \right)\left(n\right)\to (2)$$

It is clear from (1) and (2) that, $\underline{S}(\eta )$ is a fuzzy ternary subsemiring of $R.$

Now, we show that the lower approximation of a fuzzy ternary subsemiring is not a fuzzy subsemiring for SVH in general.

### Example 3.2

Let ${H}=\{0,{k},{l},{m},{n}\}$ be a ternary semiring with addition and ternary multiplication defined as $({lmn})=({lm}){n}$ given in Tables 2 and 3.

**Table 2:** Addition on $H$.

+	*0*	*k*	*l*	*m*	*n*
*0*	*0*	*k*	*l*	*m*	*n*
*k*	*k*	*l*	*m*	*k*	*k*
*l*	*l*	*m*	*k*	*l*	*l*
*m*	*m*	*k*	*l*	*m*	*n*
*n*	*n*	*k*	*l*	*n*	*n*

**Table 3:** Ternary multiplication on $H$.

	0	*k*	*l*	*m*	*n*
0	0	0	0	0	0
*k*	0	*k*	0	0	0
*l*	0	0	*l*	0	0
*m*	0	0	0	m	0
*n*	0	0	0	0	*n*

Now, let $S\hspace{0.33em}\hspace{0.33em}:\hspace{0.33em}\hspace{0.33em}H\to P(H)$ be defined as $S(0)=\{0\},S(k)=\{k\},S(l)=\{l\},S(m)=S(n)=\{m,n\},$ then $S$ is SVH.

If we let $\eta$ the fuzzy subset of $H$ given by $\eta (0)=1,\eta (k)=\eta (l)=0.5,\eta (m)=0.7,\eta (n)=0.4.$

Clearly, $\eta$ is the fuzzy subsemiring of $H.$ Then by using the definition of lower approximation, $\underline{S}(\eta )(0)=1,\underline{S}(\eta )(k)=0.5,\underline{S}(\eta )(l)=0.5,\underline{S}(\eta )(m)=0.4$ and $\underline{S}(\eta )(n)=0.4.$ This implies that $\underline{S}(\eta )$ is not a fuzzy subsemiring of $H$, because $\underline{S}(\eta )(k+l)\ge \underline{S}(\eta )(k)\wedge \underline{S}(\eta )(l)$ is not a satisfied as $\underline{S}(\eta )(k+l)=\underline{S}(\eta )(m)=0.4$ and $\underline{S}(\eta )(k)\wedge \underline{S}(\eta )(l)=0.5\wedge 0.5=0.5.$

### Theorem 3.3

If ${S}$ is a SVH and $\upeta$ is a fuzzy ternary subsemiring of ${R}$, then $\overline{{S}}(\upeta )$ is a fuzzy ternary subsemiring of ${R}$.

### Proof

Since $\eta$ is a fuzzy ternary subsemiring of $R$ , then for all $l,m,n\in R,$ $\eta (l+m)\ge \eta (l)\wedge \eta (m)$ and $\eta (lmn)\ge \eta (l)\wedge \eta (m)\wedge \eta (n).$

Consider,

$$\begin{aligned} \bar{S}\left( \eta \right)\left( {l + m} \right) & = \mathop \vee \limits_{{r \in S\left( {l + m} \right)}} \eta \left( r \right) \\ & \ge \mathop \vee \limits_{{r \in \left[ {S\left( l \right) + S\left( m \right)} \right]}} \eta \left( r \right) \\ &= \mathop \vee \limits_{{q + h \in \left[ {S\left( l \right) + S\left( m \right)} \right]}} \eta \left( {q + h} \right){\text{ take }}r = q + h{\text{ such that }}q \in S\left( l \right){\text{ and }}h \in S\left( m \right) \\ & \ge \mathop \vee \limits_{{q \in S\left( l \right),h \in S\left( m \right)}} \left\{ {\eta \left( q \right) \wedge \eta \left( h \right)} \right\} = \left\{ {\mathop \vee \limits_{{q \in S\left( l \right)}} \eta \left( q \right)} \right\} \wedge \left\{ {\mathop \vee \limits_{{h \in S\left( m \right)}} \eta \left( h \right)} \right\} \\ &= \bar{S}(\eta )(l) \wedge \bar{S}(\eta )(m). \\ \end{aligned}$$

This implies$$, \overline{S}(\eta )(l+m)\ge \overline{S}(\eta )(l)\wedge \overline{S}(\eta )(m).$$

Again consider,

$$\begin{aligned} \bar{S}\left( \eta \right)\left( {lmn} \right) & = \mathop \vee \limits_{{r \in S\left( {lmn} \right)}} \eta \left( r \right) \\ & \ge \mathop \vee \limits_{{r \in S\left( l \right)S\left( m \right)S\left( n \right)}} \eta \left( r \right) \\ & = \mathop \vee \limits_{{pqh \in S\left( l \right)S\left( m \right)S\left( n \right)}} \eta \left( {pqh} \right){\text{ take }}r = pqh{\text{such that }}p \in S\left( l \right),q \in S\left( m \right),h \in S\left( n \right) \\ & \ge \mathop \vee \limits_{{p \in S\left( l \right),q \in S\left( m \right),h \in S\left( n \right)}} \left\{ {\eta \left( p \right) \wedge \eta \left( q \right) \wedge \eta \left( h \right)} \right\} \\ & = \left\{ {\mathop \vee \limits_{{p \in S\left( l \right)}} \eta \left( p \right)} \right\} \wedge \left\{ {\mathop \vee \limits_{{q \in S\left( m \right)}} \eta \left( q \right)} \right\} \wedge \left\{ {\mathop \vee \limits_{{h \in S\left( l \right)}} \eta \left( h \right)} \right\} \\ & = \bar{S}(\eta )(l) \wedge \bar{S}(\eta )(m) \wedge \bar{S}(\eta )(n). \\ \end{aligned}$$

### Theorem 3.4

If ${S}$ is a SSVH, $\upeta$ is fuzzy left (resp. right, lateral) ideal of ${R}$, then $\underset{\_}{{S}}(\upeta )$ is a fuzzy-left (resp. right, lateral) ideal for ${R}.$

### Proof

If $\eta$ is fuzzy the left ideal of $R$ , then $\eta (l+m)\ge \eta (l)\wedge \eta (m)$ and $\eta (lmn)\ge \eta (n)$. To prove $\underline{S}(\eta )$ is the fuzzy left ideal of $R,$ we must show that.

- $$\underline{S}(\eta )(l+m)\ge \underline{S}(\eta )(l)\wedge \underline{S}(\eta )(m);$$
- $$\underline{S}(\eta )(lmn)\ge \underline{S}(\eta )(n).$$

For case (i), see Theorem Mark3.

For case (ii),

$$\begin{aligned} \underset{\raise0.3em\hbox{$\smash{\scriptscriptstyle-}$}}{S} \left( \eta \right)\left( {lmn} \right) & = \mathop \wedge \limits_{{r \in S\left( {lmn} \right)}} \eta \left( r \right){\text{ since }}S{\text{ is a }}SSVH{\text{ then}} \\ & = \mathop \wedge \limits_{{r \in S\left( l \right)S\left( m \right)S\left( n \right)}} \eta \left( r \right){\text{ take }}r = pqh{\text{ such that }}p \in S\left( l \right),q \in S\left( m \right){\text{ and }}h \in S\left( n \right) \\ &= \mathop \wedge \limits_{{pqh \in S\left( l \right)S\left( m \right)S\left( n \right)}} \eta \left( {pqh} \right) \\ &= \mathop \wedge \limits_{{p \in S\left( l \right),q \in S\left( m \right),h \in S\left( n \right)}} \eta \left( {pqh} \right){\text{ since }}\eta {\text{ is left ideal of }}R. \\ & \ge \mathop \wedge \limits_{{h \in S(n)}} \eta (h) = \underset{\raise0.3em\hbox{$\smash{\scriptscriptstyle-}$}}{S} (\eta )(n). \\ \end{aligned}$$

This implies that $\underline{S}(\eta )(lmn)\ge \underline{S}(\eta )(n)$. Similarly, we can prove for right and lateral ideal.

### Example 3.5

Let ${G}=\{0,{m},{n}\}$ be ternary semiring under addition and ternary multiplication, as shown in in Tables 4 and 5:

**Table 4:** Addition on $G$.

+	*0*	m	*n*
*0*	*0*	m	*n*
*m*	m	*m*	n
*n*	*n*	n	n

**Table 5:** Ternary multiplication on $G$.

*⋅*	*0*	*m*	*n*
*0*	*0*	0	0
*m*	*0*	*m*	*n*
*n*	*0*	*n*	*n*

Let $S\hspace{0.33em}\hspace{0.33em}:\hspace{0.33em}\hspace{0.33em}G\to P(G)$ be defined as $S(0)=\{0,n\},S(m)=\{n\},S(n)=\{m,n\}$ then $S$ is an SVH. Let $\eta$ be a fuzzy subset of $G$ given by $\eta (0)=1,\eta (m)=0.5,\eta (n)=0.8,$ then $\eta$ is the fuzzy ideal of $G.$ Therefore, by the definition of lower approximation $\underline{S}(\eta )(0)=1,\underline{S}(\eta )(m)=0.8,\underline{S}(\eta )(n)=0.5.$ This implies that $\underline{S}(\eta )$ is not a fuzzy left ideal of $G$ because $\underline{S}(\eta )(nnm)\ge \underline{S}(\eta )(m)$ is not satisfied as $\underline{S}(\eta )(nnm)=\underline{S}(\eta )(n)=0.5$ and $\underline{S}(\eta )(m)=0.8$ so, $\underline{S}(\eta )(nnm)\ngeqq \underline{S}(\eta )(m).$

### Theorem 3.6

If ${S}$ is a SVH, $\upeta$ is a fuzzy left (resp. right, lateral) ideal of ${R}$ , then $\overline{{S}}(\upeta )$ is fuzzy left (resp. right, lateral) ideal for ${R}.$

### Proof

Proof is same as Theorem 3.5.

### Theorem 3.7

If ${S}$ is a SSVH and $\upeta$ is a fuzzy semiprime ideal of ${R},$ then $\underset{\_}{{S}}(\upeta )$ is a fuzzy semiprime ideal of ${R}.$

### Proof

Because $\eta$ is the fuzzy semiprime ideal of $R$, then $\eta$ is the fuzzy ideal of $R.$ Hence, by Theorem 3.5, $\underline{S}(\eta )$ is the fuzzy ideal of $R.$ Moreover, $\eta ({l}^{3})=\eta (l)$ for all $l\in R.$

To prove that $\underline{S}(\eta )$ is a fuzzy semiprime ideal of $R$, we need to show that for all $l\in R,$ $\underline{S}(\eta )({l}^{3})=\underline{S}(\eta )(l)$

Consider,

$$\begin{aligned} \underset{\raise0.3em\hbox{$\smash{\scriptscriptstyle-}$}}{S} \left( \eta \right)\left( {l^{3} } \right) & = \mathop \wedge \limits_{{r \in S\left( {l^{3} } \right)}} \eta \left( r \right){\text{ Since }}S{\text{ is }}SSVH,{\text{ then}} \\ & = \mathop \wedge \limits_{{r \in S\left( l \right)S\left( l \right)S\left( l \right)}} \eta \left( r \right){\text{ take }}r = t^{3} {\text{ such that }}t \in S\left( l \right),{\text{then}} \\ & = \mathop \wedge \limits_{{t^{3} \in S\left( l \right)S\left( l \right)S\left( l \right)}} \eta \left( {t^{3} } \right) = \mathop \wedge \limits_{{t \in S\left( l \right)}} \eta \left( t \right) \\ &= \underset{\raise0.3em\hbox{$\smash{\scriptscriptstyle-}$}}{S} (\eta )(l). \\ \end{aligned}$$

Therefore, $\underline{S}(\eta )$ is a fuzzy semiprime ideal of $H.$

### Theorem 3.8

If ${S}$ is a SSVH and $\upeta$ is a fuzzy semiprime ideal of ${R}$, then $\overline{{S}}(\upeta )$ is the fuzzy semiprime ideal of ${R}.$

### Proof

Proof is same as Theorem 3.7.

### Theorem 3.9

If ${S}$ is a SSVH, and $\upeta$ is a fuzzy prime ideal of ${R}$, then $\underset{\_}{{S}}(\upeta )$ is the fuzzy prime ideal of ${R}.$

### Proof

Because, $\eta$ is fuzzy prime ideal of $R$ so, $\eta (lmn)=\eta (l),$ $\eta (lmn)=\eta (m)$ or $\eta (lmn)=\eta (n)$ for all $l,m,n\in R.$

Because, $\eta$ is a fuzzy ideal of $R,$ therefore by Theorem 3.5, $\underline{S}(\eta )$ is the fuzzy ideal of $R,$ we require for all $l,m,n\in R$

$$\underline{S}(\eta )(lmn)=\underline{S}(\eta )(l)$$ or $$\underline{S}(\eta )(lmn)=\underline{S}(\eta )(m)$$ or $$\underline{S}(\eta )(lmn)=\underline{S}(\eta )(n).$$

Consider,

$$\begin{aligned} \underset{\raise0.3em\hbox{$\smash{\scriptscriptstyle-}$}}{S} \left( \eta \right)\left( {lmn} \right) & = \mathop \wedge \limits_{{r \in S\left( {lmn} \right)}} \eta \left( r \right){\text{ since }}S{\text{ is a }}SSVH{\text{ then}} \\ &= \mathop \wedge \limits_{{r \in S\left( l \right)S\left( m \right)S\left( n \right)}} \eta \left( r \right){\text{ now }}r = pqh{\text{ such that }}p \in S\left( l \right),q \in S\left( m \right){\text{ and }}h \in S\left( n \right) \\ &= \mathop \wedge \limits_{{pqh \in S\left( l \right)S\left( m \right)S\left( n \right)}} \eta \left( {pqh} \right) \\ & = \mathop \wedge \limits_{{p \in S(l),q \in S(m),h \in S(n)}} \eta (pqh) \\ \end{aligned}$$

This implies, $\underline{S}(\eta )(lmn)=\underset{p\in S(l)}{\wedge }\eta (p)=\underline{S}(\eta )(l)$ or $\underline{S}(\eta )(lmn)=\underset{q\in S(m)}{\wedge }\eta (q)=\underline{S}(\eta )(m)$ or $\underline{S}(\eta )(lmn)=\underset{h\in S(n)}{\wedge }\eta (h)=\underline{S}(\eta )(n).$ Therefore, $\underline{S}(\eta )$ is the fuzzy prime ideal of $R.$

### Theorem 3.10

If ${S}$ is a SSVH and $\upeta$ is the fuzzy prime ideal of ${R},$ then $\overline{{S}}(\upeta )$ is the fuzzy prime ideal of ${R}.$

### Proof

Proof is same as Theorem 3.9.

## Approximations of $(\in ,\in \vee {\varvec{q}})$ -fuzzy ideals in ternary semirings

This section is very important because here we have discussed the lower and upper approximations of $(\in ,\in \vee q)$ -fuzzy ternary subsemirings, $(\in ,\in \vee q)$ -fuzzy ideals, $(\in ,\in \vee q)$ -fuzzy prime ideals and $(\in ,\in \vee q)$ -fuzzy semiprime ideals of ternary semirings. This section is a generaization of Sect. "[Sec Sec4]", and we also use SVH and SSVH.

### Theorem 4.1

If ${S}$ is a SSVH and $\upeta$ is an $(\in ,\in \vee {q})$ -fuzzy ternary subsemiring of ${R}$, then $\underset{\_}{{S}}(\upeta )$ is an $(\in ,\in \vee {q})$ -fuzzy ternary subsemiring of ${R}.$

### Proof

Let, ${l}_{t},{m}_{\delta },{n}_{r}\in \underline{S}(\eta )$ where $l,m,n\in R$ and $t,\delta ,r\in ({0,1}],$ $\underline{S}(\eta )(l)\ge t,\underline{S}(\eta )(m)\ge \delta$ and $\underline{S}(\eta )(n)\ge r$.

Consider,

$$\begin{aligned} \underset{\raise0.3em\hbox{$\smash{\scriptscriptstyle-}$}}{S} \left( \eta \right)\left( {l + m} \right) & = \mathop \wedge \limits_{{u \in S\left( {l + m} \right)}} \eta \left( u \right){\text{ since}},S{\text{ is }}SSVH \\ &= \mathop \wedge \limits_{{u \in \left[ {S\left( l \right) + S\left( m \right)} \right]}} \eta \left( u \right){\text{ take }}u = o + p{\text{where }}o \in S\left( l \right){\text{and }}p \in S\left( m \right) \\ &= \mathop \wedge \limits_{{\left( {o + p} \right) \in \left[ {S\left( l \right) + S\left( m \right)} \right]}} \eta \left( {o + p} \right) \\ & \ge \mathop \wedge \limits_{{\left( {o + p} \right) \in \left[ {S\left( l \right) + S\left( m \right)} \right]}} min\left\{ {\eta \left( o \right),\eta \left( p \right),0.5} \right\} \\ &= min\left\{ {\left( {\mathop \wedge \limits_{{o \in S\left( l \right)}} \eta \left( o \right)} \right),\left( {\mathop \wedge \limits_{{p \in S\left( m \right)}} \eta \left( p \right)} \right),0.5} \right\} \\ & = min\{ \underset{\raise0.3em\hbox{$\smash{\scriptscriptstyle-}$}}{S} (\eta )(l),\underset{\raise0.3em\hbox{$\smash{\scriptscriptstyle-}$}}{S} (\eta )(m),0.5\} \ge min\{ t,\delta ,0.5\} . \\ \end{aligned}$$

This implies,$$\underline{S}(\eta )(l+m)\ge \mathit{min}\{t,\delta ,0.5\}.$$

When, $\mathit{min}(t,\delta )\le 0.5$, we have, $\underline{S}(\eta )(l+m)\ge \mathit{min}(t,\delta )$ thus,$(l+m{)}_{\mathit{min}(t,\delta )}\in \underline{S}(\eta )\to (5)$

When $\mathit{min}(t,\delta )>0.5$, we have, $\underline{S}(\eta )(l+m)\ge 0.5$ therefore, $\underline{S}(\eta )(l+m)+\mathit{min}(t,\delta )>1$ then $(l+m{)}_{\mathit{min}(t,\delta )}q\underline{S}(\eta )\to (6),$ from (5) and (6)

$$(l+m{)}_{\mathit{min}(t,\delta )}\in \vee q\underline{S}(\eta )\to (7).$$

Again consider,

$$\begin{aligned} \underset{\raise0.3em\hbox{$\smash{\scriptscriptstyle-}$}}{S} \left( \eta \right)\left( {lmn} \right) & = \mathop \wedge \limits_{{u \in S\left( {lmn} \right)}} \eta \left( u \right) \\ &= \mathop \wedge \limits_{{u \in S(l)S(m)S(n))}} \eta \left( u \right){\text{ take }}u = opq{\text{where }}o \in S\left( l \right),p \in S\left( m \right),q \in S\left( n \right) \\ &= \mathop \wedge \limits_{{opq \in S\left( l \right)S\left( m \right)S\left( n \right)}} \eta \left( {opq} \right) \\ & \ge \mathop \wedge \limits_{{o \in S(l),p \in S(m),q \in S(n))}} min\left\{ {\eta \left( o \right),\eta \left( p \right),\eta \left( q \right),0.5} \right\} \\ & = min\left\{ {\left( {\mathop \wedge \limits_{{o \in S(l)}} \eta (o)} \right),\left( {\mathop \wedge \limits_{{p \in S(m)}} \eta (p)} \right),\left( {\mathop \wedge \limits_{{q \in S(m)}} \eta (q)} \right),0.5} \right\} \\ & = min\left\{ {\underset{\raise0.3em\hbox{$\smash{\scriptscriptstyle-}$}}{S} \left( \eta \right)\left( l \right),\underset{\raise0.3em\hbox{$\smash{\scriptscriptstyle-}$}}{S} \left( \eta \right)\left( m \right),\underset{\raise0.3em\hbox{$\smash{\scriptscriptstyle-}$}}{S} \left( \eta \right)\left( n \right),0.5} \right\} \\ & \ge min\{ t,\delta ,r,0.5\} . \\ \end{aligned}$$

This implies that, $\underline{S}(\eta )(lmn)\ge \mathit{min}\{t,\delta ,r,0.5\}.$ When, $\mathit{min}(t,\delta ,r)\le 0.5$ then $\underline{S}(\eta )(lmn)\ge \mathit{min}(t,\delta ,r)$ implies, $(lmn{)}_{\mathit{min}(t,\delta ,r)}\in \underline{S}(\eta )\to (8).$ When $\mathit{min}(t,\delta ,r)>0.5$, we have $\underline{S}(\eta )(lmn)\ge 0.5$ therefore, $\underline{S}(\eta )(lmn)+\mathit{min}(t,\delta ,r)>1$ then $(lmn{)}_{\mathit{min}(t,\delta ,r)}q\underline{S}(\eta )\to (9)$ from (8) and (9),$(lmn{)}_{\mathit{min}(t,\delta ,r)}\in \vee q\underline{S}(\eta )\to (10).$

It is clear from (7) and (10) that, $\underline{S}(\eta )$ is an $(\in ,\in \vee q)$ -fuzzy ternary subsemiring of $R.$

### Theorem 4.2

If ${S}$ is a SVH and $\upeta$ is a $(\in ,\in \vee {q})$ -fuzzy ternary subsemiring of ${R},$ then $\overline{{S}}(\upeta )$ is an $(\in ,\in \vee {q})$—fuzzy ternary subsemiring of ${R}.$

### Proof

Let, ${l}_{t},{m}_{\delta },{n}_{r}\in \overline{S}(\eta )$ where $l,m,n\in R$ and $t,\delta ,r\in ({0,1}],$ then $\overline{S}(\eta )(l)\ge t,\overline{S}(\eta )(m)\ge \delta$ and $\overline{S}(\eta )(n)\ge r$ .

Consider,$$\begin{aligned} \bar{S}(\eta )(l + m) & = \mathop \vee \limits_{{u \in S(l + m)}} \eta (u) \\ & \ge \mathop \vee \limits_{{u \in \left[ {S\left( l \right) + S\left( m \right)} \right]}} \eta \left( u \right) \\ &= \mathop \vee \limits_{{\left( {o + p} \right) \in \left[ {S\left( l \right) + S\left( m \right)} \right]}} \eta \left( {o + p} \right){\text{ take }}u = o + p{\text{where }}o \in S\left( l \right){\text{and }}p \in S\left( m \right) \\ & \ge \mathop \vee \limits_{{o \in S\left( l \right),p \in S\left( {m^{\prime} } \right)}} min\left\{ {\eta \left( o \right),\eta \left( p \right),0.5} \right\} \\ & = min\left\{ {\left( {\mathop \vee \limits_{{o \in S\left( l \right)}} \eta \left( o \right)} \right),\left( {\mathop \vee \limits_{{p \in S\left( m \right)}} \eta \left( p \right)} \right),0.5} \right\} \\ &= min\{ \bar{S}(\eta )(l),\bar{S}(\eta )(m),0.5\} \ge min\{ t,\delta ,0.5\} . \\ \end{aligned}$$

This implies,$$\overline{S}(\eta )(l+m)\ge \mathit{min}\{t,\delta ,0.5\}.$$

When, $\mathit{min}(t,\delta )\le 0.5$, we have, $\overline{S}(\eta )(l+m)\ge \mathit{min}(t,\delta )$ thus,$(l+m{)}_{\mathit{min}(t,\delta )}\in \overline{S}(\eta )\to (5).$

When $\mathit{min}(t,\delta )>0.5$ , we have, $\overline{S}(\eta )(l+m)\ge 0.5$ therefore, $\overline{S}(\eta )(l+m)+\mathit{min}(t,\delta )>1$ Then, $(l+m{)}_{\mathit{min}(t,\delta )}q\overline{S}(\eta )\to (6)$ from (5) and (6)$(l+m{)}_{\mathit{min}(t,\delta )}\in \vee q\overline{S}(\eta )\to (7).$

Again consider,

$$\begin{aligned} \bar{S}\left( \eta \right)\left( {lmn} \right) & = \mathop \vee \limits_{{u \in S\left( {lmn} \right)}} \eta \left( u \right) \\ & \ge \mathop \vee \limits_{{u \in S\left( l \right)S\left( m \right)S\left( n \right)}} \eta \left( u \right){\text{ take }}u = opq{\text{where }}o \in S\left( l \right),p \in S\left( m \right),q \in S\left( n \right) \\ & = \mathop \vee \limits_{{opq \in S\left( l \right)S\left( m \right)S\left( n \right)}} \eta \left( {opq} \right) \\ & \ge \mathop \vee \limits_{{o \in S(l),p \in S(m),q \in S(n))}} min\left\{ {\eta \left( o \right),\eta \left( p \right),\eta \left( q \right),0.5} \right\} \\ & = min\left\{ {\left( {\mathop \vee \limits_{{o \in S\left( l \right)}} \eta \left( o \right)} \right),\left( {\mathop \vee \limits_{{p \in S\left( m \right)}} \eta \left( p \right)} \right),\left( {\mathop \vee \limits_{{q \in S\left( m \right)}} \eta \left( q \right)} \right),0.5} \right\} \\ & = min\{ \bar{S}(\eta )(l),\bar{S}(\eta )(m),\bar{S}(\eta )(n),0.5\} \\ \end{aligned}$$

$\ge \mathit{min}\{t,\delta ,r,0.5\}$ this implies that,$\overline{S}(\eta )(lmn)\ge \mathit{min}\{t,\delta ,r,0.5\}.$

When, $\mathit{min}(t,\delta ,r)\le 0.5$ then $\overline{S}(\eta )(lmn)\ge \mathit{min}(t,\delta ,r)$, implies,$(lmn{)}_{\mathit{min}(t,\delta ,r)}\in \overline{S}(\eta )\to (8).$

When $\mathit{min}(t,\delta ,r)>0.5$ , we have $\overline{S}(\eta )(lmn)\ge 0.5$ consequently, $\overline{S}(\eta )(lmn)+\mathit{min}(t,\delta ,r)>1$ then $(lmn{)}_{\mathit{min}(t,\delta ,r)}q\overline{S}(\eta )\to (9)$ from (8) and (9)$(lmn{)}_{\mathit{min}(t,\delta ,r)}\in \vee q\overline{S}(\eta )\to (10).$

It is clear from (7) and (10), that $\overline{S}(\eta )$ is $(\in ,\in \vee q)$ -fuzzy ternary subsemiring of $R.$

### Theorem 4.3

If ${S}$ is a SSVH and $\upeta$ is an $(\in ,\in \vee {q})$ -fuzzy left (resp. right, lateral) ideal of ${R}$ , then $\overline{{S}}(\upeta )$ is an $(\in ,\in \vee {q})$ -fuzzy left (resp. right, lateral) ideal for ${R}.$

### Proof

Since $\eta$ is an $(\in ,\in \vee q)$ -fuzzy left ideal of $H,$ to prove that $\overline{S}(\eta )$ is $(\in ,\in \vee q)$-fuzzy left ideal of $R,$ we want to show that following conditions.

(i) $${l}_{t},{m}_{r}\in \overline{S}(\eta )\to (l+m{)}_{\mathit{min}(t,\delta )}\in \vee q\overline{S}(\eta )$$
(ii) $${l}_{r}\in \overline{S}(\eta )$$ and $$m,n\in R\to (mnl{)}_{r}\in \vee q\overline{S}(\eta )$$

Case (i): see Theorem 4.2.

Case (ii): Let ${l}_{r}\in \overline{S}(\eta )$ and $m,n\in R,$ then $\overline{S}(\eta )(l)\ge r$

Consider,

$$\begin{aligned} \bar{S}\left( \eta \right)\left( {mnl} \right) & = \mathop \vee \limits_{{u \in S\left( {mnl} \right)}} \left( \eta \right)\left( u \right); \\ & = \mathop \vee \limits_{{u \in S\left( m \right)S\left( n \right)S\left( l \right)}} \left( \eta \right)\left( u \right){\text{ take }}u = opq{\text{ where }}o \in S\left( m \right),p \in S\left( n \right),q \in S\left( l \right); \\ & = \mathop \vee \limits_{{u \in S\left( m \right)S\left( n \right)S\left( l \right)}} \eta \left( {opq} \right) \ge \mathop \vee \limits_{{q \in S\left( l \right)}} min\left\{ {\eta \left( q \right),0.5} \right\} \\ & = min\left\{ {\mathop \vee \limits_{{q \in S\left( l \right)}} \eta \left( q \right),0.5} \right\} = min\left\{ {\bar{S}\left( \eta \right)\left( l \right),0.5} \right\} \\ &\ge min(t,0.5). \\ \end{aligned}$$

When, $r\le 0.5,$ we have $\overline{S}(\eta )(mnl)\ge r$ this implies,$(mnl{)}_{r}\overline{S}(\eta )\to (1).$

When, $r>0.5,$ we have $\overline{S}(\eta )(mnl)+r>1$ which implies that, $(mnl{)}_{r}\in \vee q\overline{S}(\eta )\to (2)$ from (1) and (2) which implies that, $(mnl{)}_{r}\in \vee q\overline{S}(\eta )$ . Hence, $\overline{S}(\eta )$ is a $(\in ,\in \vee q)$ -fuzzy left ideal. Similarly, we can prove the lateral and right ideals of $R.$

### Theorem 4.4

If ${S}$ is a SSVH and $\upeta$ is an $(\in ,\in \vee {q})$ -fuzzy left (resp. right, lateral) ideal of ${R},$ then $\underset{\_}{{S}}(\upeta )$ is an $(\in ,\in \vee {q})$ -fuzzy left (resp. right, lateral) ideal for ${R}.$

### Proof

Proof is same as Theorem 4.3.

### Theorem 4.5

If ${S}$ is a SSVH and $\upeta$ is an $(\in ,\in \vee {q})$ -fuzzy semiprime ideal of ${R},$ then $\overline{{S}}(\upeta )$ is a $(\in ,\in \vee {q})$ -fuzzy semiprime ideal of ${R}.$

### Proof

Let $({l}^{3}{)}_{t}\in \overline{S}(\eta )$ for all $l\in R$ , $t\in ({0,1}]$ then $\overline{S}(\eta )({l}^{3})\ge t$ .

Consider,

$$\begin{aligned} \bar{S}\left( \eta \right)\left( l \right) & = \mathop \vee \limits_{{u \in S\left( l \right)}} \eta \left( u \right) \\ & \ge \mathop \vee \limits_{{u^{3} \in S(l)^{3} }} min\left\{ {\eta \left( {u^{3} } \right),0.5} \right\} \\ & = \mathop \vee \limits_{{u^{3} \in S\left( {l^{3} } \right)}} min\left\{ {\eta \left( {u^{3} } \right),0.5} \right\} \\ &= min\left\{ {\mathop \vee \limits_{{u^{3} \in S\left( {l^{3} } \right)}} \eta \left( {u^{3} } \right),0.5} \right\} \\ & = min\{ \bar{S}\eta (l^{3} ),0.5\} \ge min(t,0.5). \\ \end{aligned}$$

When $t\le 0.5$ this implies, $\overline{S}(\eta )(l)\ge t$ then ${l}_{t}\in$ $\overline{S}(\eta ).$ When, $t>0.5,$ we have $\overline{S}(\eta )(l)\ge 0.5$ thus, $\overline{S}(\eta )(l)+t>1$ then ${l}_{t}q\overline{S}(\eta )$ therefore, we get ${l}_{t}\in \vee q\overline{S}(\eta ).$ Therefore, $\overline{S}(\eta )$ is an $(\in ,\in \vee q)$ -fuzzy semiprime ideal for $R.$

### Theorem 4.6

If ${S}$ is a SSVH with $\upeta$ is an $(\in ,\in \vee {q})$ -fuzzy semiprime ideal of ${H}$ then $\underset{\_}{{S}}(\upeta )$ is a $(\in ,\in \vee {q})$—fuzzy semiprime ideal of H.

### Proof

Proof is same as Theorem 4.5.

### Theorem 4.7

If ${S}$ is a SSVH and $\upeta$ is an $(\in ,\in \vee {q})$ -fuzzy prime ideal of ${R},$ then $\overline{{S}}(\upeta )$ is a $(\in ,\in \vee {q})$ -fuzzy prime ideal of ${R}.$

### Proof

Since $\eta$ is an $(\in ,\in \vee q)$ -fuzzy prime ideal of $R$ , so $\eta$ is an $(\in ,\in \vee q)$ -fuzzy ideal of $R.$ Thus by Theorem 4.3, $\overline{S}(\eta )$ is $(\in ,\in \vee q)$ -fuzzy ideal of $R.$ Further by Theorem 2.14,$\mathit{max}\{\eta (l),\eta (m),\eta (n)\}\ge \mathit{min}\{\eta (lmn),0.5\}$

Let $(lmn{)}_{t}\in \overline{S}(\eta )$ then $\overline{S}(\eta )(lmn)\ge t$ for $l,m,n\in H$ and $t\in ({0,1}]$

Consider,

$$\begin{aligned} \bar{S}\left( \eta \right)\left( l \right) \vee \bar{S}\left( \eta \right)\left( m \right) \vee \bar{S}\left( \eta \right)\left( n \right) & = \left\{ {\mathop \vee \limits_{{o \in S\left( l \right)}} \eta \left( o \right)} \right\} \vee \left\{ {\mathop \vee \limits_{{p \in S\left( m \right)}} \eta \left( p \right)} \right\} \vee \left\{ {\mathop \vee \limits_{{q \in S\left( n \right)}} \eta \left( q \right)} \right\} \\ &= \mathop \vee \limits_{{o \in S\left( l \right),p \in S\left( m \right),q \in S\left( n \right)}} \left\{ {\eta \left( a \right) \vee \eta \left( b \right) \vee \eta \left( c \right)} \right\} \\ &\ge \mathop \vee \limits_{{o \in S\left( l \right),p \in S\left( m \right),q \in S\left( n \right)}} \left\{ {\eta \left( {opq} \right) \wedge 0.5} \right\} \\ & = \mathop \vee \limits_{{opq \in S\left( {lmn} \right)}} \left\{ {\eta \left( {opq} \right) \wedge 0.5} \right\} \\ & = \bar{S}(\eta )(lmn) \wedge 0.5 \ge \mathop {min}\limits^{{\mathop {\phantom{0}}\limits_{{\phantom{0}}} }} (t,0.5) \\ \end{aligned}$$

When $t\le 0.5$ we have $\overline{S}(\eta )(l)\vee \overline{S}(\eta )(m)\vee \overline{S}(\eta )(n)\ge t$ then, $\overline{S}(\eta )(l)\ge t$ or $\overline{S}(\eta )(m)\ge t$ or $\overline{S}(\eta )(n)\ge t.$ Therefore, ${l}_{t}\in \overline{S}(\eta )$ or ${m}_{t}\in \overline{S}(\eta )$ or ${n}_{t}\in \overline{S}(\eta )$ .When $t>0.5$ we have $\overline{S}(\eta )(l)\vee \overline{S}(\eta )(m)\vee \overline{S}(\eta )(n)\ge 0.5$, therefore $\overline{S}(\eta )(l)\ge 0.5$ or $\overline{S}(\eta )(m)\ge 0.5$ or $\overline{S}(\eta )(n)\ge 0.5$ implies, $\overline{S}(\eta )(l)+t>1$ or $\overline{S}(\eta )(l)+t\ge 1$ or $\overline{S}(\eta )(l)+t\ge 1$

Hence, ${l}_{r}q\overline{S}(\eta )$ or ${m}_{t}q\overline{S}(\eta )$ or ${n}_{t}q\overline{S}(\eta )$. Therefore, ${l}_{t}\in \vee q\overline{S}(\eta )$ or ${m}_{t}\in \vee q\overline{S}(\eta )$ or ${n}_{t}\in \vee q\overline{S}(\eta )$ .

Therefore, $\overline{S}(\eta )$ is an $(\in ,\in \vee q)$ -fuzzy prime ideal of $R.$

### Theorem 4.8

If ${S}$ is a SSVH and $\upeta$ is an $(\in ,\in \vee {q})$ -fuzzy prime ideal of ${R},$ then $\underset{\_}{{S}}(\upeta )$ is an $(\in ,\in \vee {q})$ -fuzzy prime ideal of ${R}.$

### Proof

Proof is same as above Theorem 4.7.

## Comparative study

The work of Ref.^27^ introduced the roughness in $(\in ,\in \vee q)$-fuzzy ideals of semirings and Ref.^29^ worked on roughness of $(\in ,\in \vee q)$-fuzzy ideals of ternary semigroups. By continuing this work, we studied the roughness of $(\in ,\in \vee q)$-fuzzy ideals of ternary semirings by introducing two operations. Our methodology is a generalized form of Refs.^27,29^ and is better because the sets of negative integers, negative rational numbers and negative real numbers are not semirings. However, these are ternary semirings and the applied technique is useful for these structures.

## Conclusions

In this study, we generalize Refs.^27^ and^29^ to a new algebraic framework. We introduced new definitions for SVH and SSVH using a ternary operation. We observe that the lower approximations of the fuzzy ternary subsemiring (resp. fuzzy ideals) by applying SVH is fuzzy ternary subsemiring (resp. fuzzy ideals) in $R$. The upper approximations of the fuzzy ternary subsemiring (resp. fuzzy ideals) using SSVH are fuzzy ternary subsemirings (resp. fuzzy ideals). We also prove using an example that the lower approximation of a fuzzy ternary subsemiring (resp. fuzzy ideal) is not fuzzy ternary subsemiring (resp. fuzzy ideal) for the SVH. It is also proven that the approximations of the fuzzy prime (resp. semiprime) ideals using the SSVH are fuzzy primes (resp. semiprime) ideals.

We see that the lower approximations of an $(\in ,\in \vee q)$ -fuzzy ternary subsemiring using SSVH are $(\in ,\in \vee q)$ -fuzzy ternary subsemiring and upper approxiamtions of an $(\in ,\in \vee q)$-fuzzy ternary subsemiring using SVH is an $(\in ,\in \vee q)$ -fuzzy ternary subsemiring. It is also proved that the approximations of $(\in ,\in \vee q)$ -fuzzy ideals using SSVH are $(\in ,\in \vee q)$ -fuzzy ideals.

We consider that in the near future, the form of roughness obtained by applying set admired maps will be drawn out to alternative algebraic arrangements.

## Data Availability

The datasets used and analyzed during the study are available from the corresponding author upon reasonable request.
