# Author Correction: Generalized roughness of three dimensional (∈, ∈ ∨ *q*) fuzzy ideals in terms of set‑valued homomorphism

**DOI:** 10.1038/s41598-024-75674-w

**Published:** 2024-10-22

**Authors:** Shahida Bashir, Rabia Mazhar, Nasreen Kausar, Saziye Yaman, Syed Suleman Ali, Muneeb Ul Hassan Afzal

**Affiliations:** 1 Department of Mathematics, University of Gujrat, Gujrat, 50700 Pakistan; 2 Department of Mathematics, Faculty of Arts and Science, Yildiz Technical University, 34220 Esenler, Istanbul, Turkey; 3 Department of Liberal Arts, American University of the Middle East, 54200 Egaila, Kuwait; 4 Department of Mechanical Engineering, University of Gujrat, Gujrat, 50700 Pakistan

Correction to: *Scientific Reports*10.1038/s41598-024-62207-8, published online 29 May 2024

The original version of this Article contained errors.

In the Abstract,

“The objective of this study is to generalize the roughness of a fuzzy set-in three-dimensional structure by introducing ternary multiplication.”

now reads:

“The objective of this study is to generalize the roughness of a fuzzy set in three-dimensional structure by introducing ternary multiplication.”

Furthermore, the ‘Preliminaries’ section, contained errors.

Under the subheading ’Definition 2.5’.

“A fuzzy ideal $$\eta$$ of $$R$$ is called a semiprime if $$\eta (rrr)\ge \eta (r)$$ and prime if $$\eta (opq)=\eta (o)$$ or $$\eta (opq)=\eta (p)$$ or $$\eta (opq)=\eta (q)$$ for all $$o,p,q,r\in R$$ (2) $$\eta (opq)\ge \eta (q)$$$$($$ resp. $$\eta (opq)\ge \eta (o)$$ , $$\eta (opq)\ge \eta (p));$$”

now reads:

“A fuzzy ideal $\eta$ of $R$ is called a semiprime if $\eta (rrr)\ge \eta (r)$ and prime if $\eta (opq)=\eta (o)$ or $\eta (opq)=\eta (p)$ or $\eta (opq)=\eta (q)$ for all $o,p,q,r\in R$”

Additionally, ‘Definition 2.7’:

“An $(\in ,\in \vee q)$ - fuzzy left (resp. right and lateral), the ideal $\eta$*of*$R$ is defined as for all $l,m,n\in R$ and $r,t\in (\text{0,1}]"$

now reads:

“An $(\in ,\in \vee q)$ - fuzzy left (resp. right and lateral) the ideal $\eta$*of*$R$ is defined as for all $l,m,n\in R$ and $r,t\in (\text{0,1}]$”

And, in Definition 2.16, a lower bar was typeset incorrectly. The definition now reads:

“For every $$x\in R,$$ we define fuzzy subsets $$\underline{{S(\eta )}}(l)=\underset{n\in S(l)}{\wedge }\eta (n)$$ and $$\overline{S(\eta )}(l)=\underset{n\in S(l)}{\vee }\eta (n).$$$$\underline{{S(\eta )}}$$ is the lower approximation and $$\overline{S(\eta )}$$ is the upper approximation of $$\eta$$ with respect to mapping $$S$$. Combination $$(\underline{{S(\eta )}},\overline{S(\eta )})$$ is called rough fuzzy subset of $$R$$ if $$\underline{{S(\eta )}}=\overline{S(\eta )}.$$

In addition, section ‘Approximations of fuzzy ideals in ternary semirings’ contained errors.

In Proof of ‘Theorem 3.4’,

“For case (i), see Theorem Mark3.”

now reads:

“For case (i), see Theorem 1.”

In Proof of Theorem 3.6,

“Proof is same as Theorem 3.5.”

now reads:

“Proof is same as Theorem 3.4.”

In Proof of Theorem 3.7,

“Because $\eta$ is the fuzzy semiprime ideal of R, then $\eta$ is the fuzzy ideal of R. Hence, by Theorem 3.5, S($\eta$*)* is the fuzzy ideal of R*.*”

now reads:

“Because $\eta$ is the fuzzy semiprime ideal of R, then $\eta$ is the fuzzy ideal of R. Hence, by Theorem 3.4, S($\eta$*)* is the fuzzy ideal of R*.*”

Finally, in Proof of Theorem 3.9,

“Because, $\eta$ is a fuzzy ideal of R, therefore by Theorem 3.5, S($\eta$*)* is the fuzzy ideal of R, we require for all l, m, n ∈ R”

now reads:

“Because, $\eta$ is a fuzzy ideal of R, therefore by Theorem 3.4, S($\eta$*)* is the fuzzy ideal of R, we require for all l, m, n ∈ R”

The original article has been corrected.

